# The Controversy, Challenges, and Potential Benefits of Putative Female Germline Stem Cells Research in Mammals

**DOI:** 10.1155/2016/1728278

**Published:** 2015-12-15

**Authors:** Zezheng Pan, Mengli Sun, Xia Liang, Jia Li, Fangyue Zhou, Zhisheng Zhong, Yuehui Zheng

**Affiliations:** ^1^Medical Teaching Laboratory Center, Jiangxi Medical College, Nanchang University, Nanchang 330031, China; ^2^Faculty of Basic Medical Science, Jiangxi Medical College, Nanchang University, Nanchang 330031, China; ^3^College of Life Science, Chinese PLA General Hospital, Beijing 100039, China; ^4^The Second Affiliated Hospital of Nanchang University, Nanchang University, Nanchang 330006, China

## Abstract

The conventional view is that female mammals lose their ability to generate new germ cells after birth. However, in recent years, researchers have successfully isolated and cultured a type of germ cell from postnatal ovaries in a variety of mammalian species that have the abilities of self-proliferation and differentiation into oocytes, and this finding indicates that putative germline stem cells maybe exist in the postnatal mammalian ovaries. Herein, we review the research history and discovery of putative female germline stem cells, the concept that putative germline stem cells exist in the postnatal mammalian ovary, and the research progress, challenge, and application of putative germline stem cells in recent years.

## 1. Introduction

In most female vertebrates embryo stages, part of the blastula cells can form primordial germ cells (PGCs) by germ cell determination (GCD) under some signal induction. Along with the embryonic development, PGCs gradually migrate from the gut, yolk sac, or allantoic base to the genital ridge by amoebic movement. In the genital ridge, PGCs and mesoblastema coform the ovary. Under the regulation of external signal molecules, PGCs can differentiate into oogonia. Next, oogonia propagate rapidly by mitosis and migrate into the ovarian cortex at the same time. Finally, oogonia enter meiosis and differentiate into primary oocytes. These primary oocytes are surrounded by a layer of granular cells and form the primitive follicle, but only a fraction of primitive follicles can develop into mature follicles and ultimately undergo ovulation.

Spermatogonial stem cells (SSCs) in male mammalian testes can constantly proliferate and differentiate to maintain persistent spermatogenesis, which allows male mammals to have a longer reproductive age than female mammals [1, 2]. However, in female mammals, the conventional viewpoint suggests that the proliferation of ovarian germ cells only occurs during the fetal period. At or shortly after birth, the propagation of oogonium ceased and began to differentiate into the primary oocytes. Hence, the number of germ cells in ovaries is fixed in postnatal ovaries. As the consumption of ovulation in reproduction cycle, the germ cells will gradually decrease until exhaustion. In other words, there are no putative germline stem cells (GSCs) existing in the postnatal mammalian ovary which can produce new oocytes to replenish the consumption of ovulation. The primordial follicle will be eventually consumed due to the lack of renewable germ cells. This seems to be a valid explanation for the shorter gestational age of mammalian females than males. In recent decades whether germ cell regeneration exists in the postnatal mammalian ovaries has become controversial. The prevailing view that there is no oogenesis in the postnatal mammalian ovaries had not been challenged until recent years, when the putative GSCs were discovered.

## 2. The Research History of Putative GSCs in Animal Ovaries

### 2.1. Definition of Putative GSCs in the Ovaries

Stem cells are a type of original undifferentiated cells that possess the ability of pluripotency and self-replication, which are characterized with round or oval shape, small cell with relatively large nucleus, and high telomerase activity. The strictest definition of a stem cell requires that it possesses two properties: self-renewal, the ability to go through indefinite cell division while maintaining an undifferentiated state, and totipotency or pluripotency, which is the ability to differentiate into embryonic or specialized cell types. Stem cells can be classified into embryonic stem cells (ESCs) and adult stem cells according to the different developmental stage, and adult stem cells can be further classified into somatic stem cells and GSCs.

GSCs are a unique cell population contributing to the production of gametes. As one kind of GSCs, SSCs are well known for the unique cells contributing to spermatogenesis during adulthood in male mammals [2, 3]. In females, the germ cells lineage includes a wide spectrum of cells ranging from PGCs, oogonia, primary oocytes, and secondary oocytes to eggs. In all cells lineage, however, there are only PGCs and oogonia possess the ability of mitosis to self-propagate. Recently, neo-oogenesis or follicular renewal was observed in adulthood mammals [4, 5], and the new oocytes were considered to be derived from GSCs which located in surface epithelium of ovaries. This kind of GSCs is referred to as the putative female germline stem cells (FGSCs) or ovarian germline stem cells (OGSCs). The concept of FGSCs or OGSCs in mammals most likely originates from Johnson and his team, whose study suggested the existence of proliferative germ cells that sustain oogenesis and follicle production in the postnatal mice ovaries [4]. If putative FGSCs can be identified as one kind of GSCs, they must have characteristics of self-renewal and pluripotency, being similar to SSCs in morphology, growth pattern, and function. In addition, putative FGSCs should also provide the germline cells special-markers to avoid being treated with other stem cells in ovaries.

### 2.2. FGSCs Exist in the Ovaries of Invertebrates and Lower Vertebrates

There are several species in which GSCs were found in their postnatal ovaries. Invertebrates, such as* Drosophila*, have been extensively used to reproductive study because their germline is a suitable model of stem cells research [6]. The advantages of* Drosophila* model include the genetically tractable, the sterility caused by GSCs absence is easily detected, and the stem cells can be simply identified by gene markers. GSCs in* Drosophila* ovaries are derived from a population of PGCs, which can maintain self-renewal and replenish new oocytes into the follicle pool by asymmetric division, therefore providing a persistent supply of germ cells throughout reproductive life span [7, 8]. Previously, Lim et al. [9] adopted a fluorescence-activated cell sorting to separate and purify a mass of undifferentiated, stem cell-like germline cells from adult* Drosophila* ovaries. More recently, some studies have also reported the mechanism about the maintenance and development of FGSCs in* Drosophila *[10–12].

In addition, FGSCs have also been discovered in other invertebrates and lower vertebrates, such as* C. elegans* and teleost fish. Like in* Drosophila*, the distal tip cell (DTC) in* C. elegans* gonad forms a stem cells niche that controls the decision of germline self-renewal and differentiation. Studies showed that the DTC governs division and development of germline by Notch signaling pathway [13, 14]. Angelo and Van Gilst [15] also found that* C. elegans* can protect its reproductive ability and has a prolonged reproductive age after enduring starvation because GSCs can generate new germ cells when the food source is recovered. Nakamura et al. [16] applied transgenic technology and clonal analysis methods to identify ovarian epithelial cells in transgenic adult teleost fish. They found a type of mitotic cell that can develop into a germ cell, mature into an egg, and, eventually, generate offspring. Therefore, Nakamura et al. concluded that a type of GSCs exists with oogonial characteristics in the ovarian beam. Subsequently, based on Neo and DsRe's research, Wong et al. [17] constructed a double-transgenic zebrafish model. Then FGSCs were isolated from the ovaries of the zebrafish model and cultured for 3–6 weeks under G418 selection. Finally, the DsRed positive cells were transplanted into 2-week-old juvenile zebrafish that had no endogenous germ cells due to pretreatment. Two weeks after transplantation, the DsRed positive cells were detected in the genital glands of the recipients. The recipients were able to reach sexual maturity and reproduced normal offspring after mating with wild-type zebrafish. The transplant results showed that FGSCs are fully capable of generating functional gametes after relocation into the genital gland niche.

### 2.3. Discovery of Putative FGSCs in the Postnatal Mammalian Ovaries

A variety of stem cell types in multiple mammalian organs and tissues, such as neural stem cells, hematopoietic stem cells, cancer stem cells, ESCs, mesenchymal stem cells, and SSCs, have been discovered in recent years. Since so many stem cells can be identified in such mammals, why would FGSCs not be present in the ovaries of postnatal mammals?

In 2004, Johnson et al. [4] measured the number of follicular atresia cases in adult mice, and the results showed that the number of atretic follicles was more than the reductive number of nonatretic follicles. In theory, the follicle number is supposed to decrease due to follicular atresia; thus, it is implied that there exists the phenomenon of neo-oogenesis in adulthood mice. Further, by histologic analysis, a few populations of ovoid cells in the ovarian surface epithelium (OSE) of pubescent mice, which is not surrounded by squamous epithelial cells and does not have the morphological structure of any other type of follicle, were identified. Dual-immunofluorescence analysis showed that these cells coexpressed the germ cell-specific marker Mvh and DNA proliferation marker BrdU. Based on the above results, Johnson et al. proposed for first time that the ovoid cells in the OSE may be putative FGSCs and the source of ongoing oogenesis during reproductive span. This discovery challenged the prevailing viewpoint in the reproductive research field that no putative FGSCs exist in the postnatal mammalian ovary. Further, Johnson et al. [18] transplanted bone marrow-derived cells from wild-type mice to infertile mice, and both immature oocytes and follicles were detected in the ovary after 2 months. These results indicated that bone marrow may be a potential source of putative FGCSs. Adopting immunological markers and other methods, Kerr et al. [19] precisely calculated the number of ovarian follicles in mice at different ages, and the results showed that there is no significant decline for the average number of follicles during the period from 7 to 100 days after birth. Considering that postnatal mice reach sexual maturity in about 8 weeks, which results in consumption of a portion of the follicle pool by ovulation, Kerr et al.'s experiments suggested that there is an oocyte supplementation mechanism in postnatal mice. Another study also revealed that atrophied ovaries of aged mice were shown to perform oogenesis when these ovaries were placed in the ovarian environment of adolescent mice [20], which implied that the oocyte supplementation mechanism may be systematically regulated by sero-factors, at least in part. The above results again indicated that a follicle supplementation mechanism exists in the ovaries of adult mice. In addition, other studies have shown that the cells derived from the ovarian cortex possessed a proliferation activity when they were cultured in vitro, and many kinds of stem and/or germ cell markers, such as Oct4, Mvh, SSEA-1, and SCF-R, were detected in those cells [20–22].

**Figure 1:**
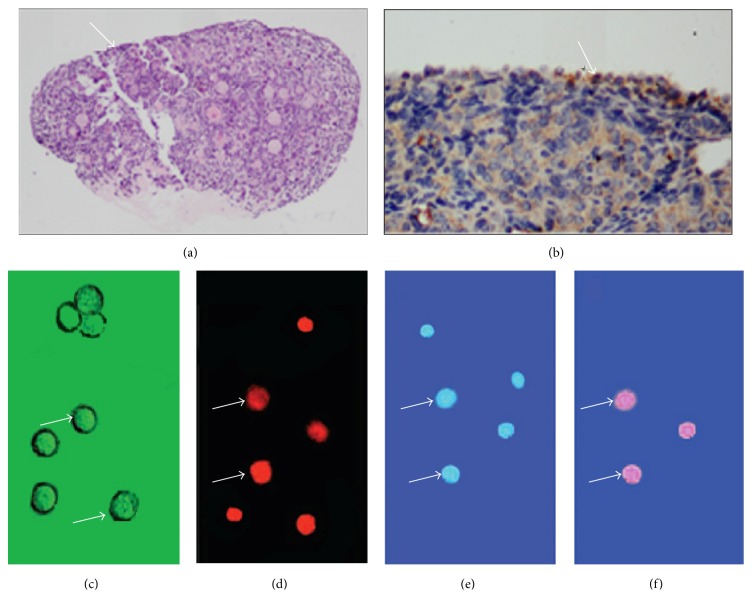
The isolation and identification of putative FGSCs. (a) H&E stained from histological ovaries sections: putative FGSCs have been considered to be mainly localized in ovarian cortex. (b) IHC results: putative FGSCs were detected by stem cell marker Oct-4 antibody. (c) Putative FGSCs were isolated by primary culture method. Arrows: the freshly isolated putative FGSCs. (d) Arrows: the isolated MVH^+^ putative FGSCs. (e) Green: the isolated BrdU^+^ cells. (f) The merger of (d) and (e) by dual immunofluorescence.

Although the above studies are consistent with the hypothesis that that follicle pool can be replenished in postnatal mammals, there is still no direct evidence that putative FGSCs exist in the postnatal ovary. However, using an immune magnetic bead cell sorting technique, Zou et al. [23] isolated and purified putative GSCs from postnatal day 5 and day 42 C57BL mice ovaries by using an antibody against the germline specific marker Mvh protein. These cells measuring 15–20 *μ*m in diameter expressed pluripotency and germ cell markers as well as a normal karyotype, ovoid in shape. This type of cells was BrdU positive, had proliferation in clusters, detected expression of alkali phosphatase and telomerase, and underwent passage over 80 times. More interestingly, morphologically normal follicles and oogenesis were observed in ovarian transplant recipients when those cells marked with GFP had been transplanted into infertile mice model. And almost 80% of the infertile mice restored fertility when they mated with wild-type males; however, there are only one-third of the offspring expressing GFP. The research by Zou and colleagues provided a key evidence to support the existence of putative FGSCs in postnatal mammalian ovaries, and these findings challenged the prevailing viewpoint that putative GSCs do not exist in postnatal mammalian ovaries and may be expected to expand a new field in stem cell research (Figure 1).

## 3. The Controversy of Putative GSCs Existing in the Postnatal Mammalian Ovaries

In 1951, Green and Zuckerman [24] first separated the ovaries of 12 viripotent rhesus monkeys and calculated the average total quantity of oocytes in different stages of the menstrual cycle. No statistically significant differences in the average number of oocytes between menarche, midcycle, and late menses were found. Therefore, Green and Zuckerman proposed that germ cells exist in female mammals after birth but remain in a status of duplication arrest and that, after menarche, some germ cells exit from duplication arrest during every menstrual cycle. Consequently, the quantity of ova is stable, at least during a particular reproductive period. Later, using adult human ovaries, Liu et al. [25] took the approach of analyzing the expression of marker genes required for neo-oogenesis. The results showed that active meiosis, neo-oogenesis, and GSCs are unlikely to exist in normal adult human ovaries. The above findings are generally accepted by some researchers in the reproductive field and were validated by more experiments [26, 27].

In the reproductive field, the validity of research results that support the existence of putative FGSCs in postnatal mammals is still debated. To validate Johnson et al.'s hypothesis that putative FGSCs are derived from marrow [18], Eggan et al. [28] transplanted marrow cells into oocyte-depleted mice to search putative FGSCs; however, it was not to find the evidence that putative FGSCs originated from bone marrow. For the results of Zou et al. experiment [23], there are also some arguments for the validity of results. For instance, isolation of putative FGSCs depends on selection by presumed germline specific markers. The works by Zou et al. are based on the assumption that Mvh is a germline specific marker; however, the research also showed that Mvh is also overexpressed in epithelial ovarian cancer, and it may be served as a valuable marker of tumorigenesis [29]. What evidence do we have that the isolated cells are more closely related to putative FGSCs than to epithelial ovarian cancer or their precursors? A growing evidence also demonstrates that oocytes have robust capacity for DNA double-strand damage repair [30]; then, maybe there is proliferation markers such as BrdU that be incorporated into oocytes DNA repair rather than cellular DNA replication. In addition, why had not all infertility mice recovered fertility, and why were only 1/3 of offspring GFP positive? Zhang et al. [31] used a Rosa26rbw/+; Ddx4-Cre transgenic mouse model to track the proliferation and differentiation of Mvh positive cells in ovaries by examining endogenous inheritance patterns, and the results of the live-cell imaging and folliculogenesis experiments showed that the Mvh positive cells in the postnatal ovary did not proliferate or differentiate into oocytes. Therefore, they suspected that no Mvh positive putative GSCs with proliferative activity exist in the postnatal mice ovaries. By means of lineage marker tracking, Lei and Spradling [32] discovered that the development of primordial follicles in ovaries is highly stable in order to maintain oogenesis and that adult mice do not have putative GSCs in either an active or a resting state. Later, Yuan et al. [33] also failed to find the putative GSCs in the ovary of postnatal mice and macaques. There is still no direct evidence to support neo-oogenesis in postnatal mammals.

Although previous studies cannot explain the phenomenon that follicle pool is replenished in adult mammalian ovaries, whether putative FGSCs contribute to the postnatal oogenesis is still controversy. Part of reproductive scholars remain hesitant to accept the fact that there are putative FGSCs in postnatal mammalian ovaries. One of the main reasons which is the key demonstration that putative FGSCs propagate and develop into follicle under normal living mice ovaries has not been still proved. The paper by Zou et al. [23] purporting to form follicles and generate offspring from putative FGSCs of postnatal mice may occur only in experiment conditions, and it may lead to cell behavior change because there are some cellular factors to be added to FGSCs medium. Until now, no one has replicated these experiments and proved that FGSCs contribute to form follicles in a physiological context. It cannot rule out a mixture of oocytes and other cells in FGSCs, with the oocytes being responsible for the generation of the offspring. More crucially, the offspring were generated only from FGSCs isolated from neonatal ovaries, not adult or even elderly ovaries. Thus, it is necessary to present the new evidences before putative FGSCs can be convinced in postnatal mammals.

## 4. The Latest Advances of Putative FGSCs in Mammals

### 4.1. Research Advancements regarding Putative GSCs in the Mice Ovaries

In order to dispel the doubts regarding the existence of putative GSCs in postnatal mammalian ovaries, Zou et al. [34] attempted to utilize another germline cell-specific protein Fragilis to isolate putative FGSCs in postnatal mice. As a transmembrane protein, Fragilis possesses an extramembrane fragment that can bind to an antibody. Using immunofluorescence assays and magnetic sorting technique, the authors successfully isolated the cells, which are the same as putative FGSCs purified by Mvh protein. Interestingly, compared with using Mvh protein, the efficiency of putative FGSCs purification was remarkably enhanced when Fragilis protein was used for isolation.

In the study to identify and track putative FSGCs, Pacchiarotti et al. [35] utilized a transgenic mouse model expressing GFP under the control of Oct-4 promoter. Oct-4 is expressed in different stages of germ cells, which can be used to visualize GFP positive cells [36]. After primary cell isolation, putative FGSCs as detected by GFP were sorted and cultured on mouse embryonic fibroblast (MEF) feeder. GFP positive cells could form flat colony, were stained positive for germ cell markers GCNA and c-Kit and pluripotent markers Oct-4 and Nanog, and maintained the telomerase activity and normal karyotype after more than 20 passages. A few cells in each colony become large and make individual round-shaped cells with different sizes. The authors speculate that these cells might be primary oocytes that have been generated from putative FGSCs colony. The results indicate that putative FGSCs might have been spontaneously differentiated into primary oocytes. More interestingly, a primordial follicle-like structure containing the oocyte-like cell in the middle and a layer of granulose cells around it was formed when these cells were aggregated with granulosa cells. In conclusion, Pacchiarotti et al. further demonstrate the presence of putative FGSCs in postnatal mouse ovaries with the ability to self-renew and differentiate to oocyte-like cells.

Hu et al. [37] also successfully isolated a type of cell with ESCs characteristics from the ovaries of neonatal and adult mice and were able to differentiate those cells into an oocyte-like structure in vitro. Currently, there is a robust technique for isolating putative FGSCs from mammalian ovaries. Based on the research of Hu et al. and others, Woods and Tilly [38] established handling procedures for putative FGSCs, including procedures for separation, culture, identification, and transplantation.

At present, putative FGSCs were discovered in postnatal mice ovaries and can be cultured in vitro. Cell therapy has been considered to recover some organs and tissues function in diseases. To this end, Terraciano et al. [39] transplanted FGSCs into cisplatin-treated wild-type recipients for 7–14 days, and the results showed that the number of follicles with apparent normal structure had a significant increase compared to control. This study suggested potential therapeutic effects for ovarian dysfunction by intraovarian microinjection of FGSCs.

Subject to the ethics issues, pluripotent stem cells such as ESCs and embryonic germ cells derived from early embryos are limited to application in clinical cell-based therapies. For overcoming the above defect in clinical application, Wang et al. [40] isolated FGSCs from neonatal and prepubertal mice ovaries and cultured FGSCs until the cells proliferated stably. Then, FGSCs were cultured in ESC-specific medium, and the results showed that female embryonic stem-like cells (fESLCs) were generated within 1 month and shared certain properties of ESCs. The findings suggest that FGSCs can be converted to fESLCs under certain culture conditions and may provide a foundation for clinical regenerative applications.

### 4.2. Research Advancements regarding Putative FGSCs in Other Mammalian Species

In addition to isolating putative FGSCs from mouse ovaries, researchers have also attempted to establish putative FGSCs from other postnatal mammalian species. Zhou et al. [41] established a new putative FGSCs line by germline cell marker Fragilis from postnatal 5-day-old rats, which showed a normal karyotype, high telomerase activity, and a consistent gene pattern of PGCs after 1 year of culture. The new putative FGSCs line could differentiate into oocytes when cultured in vitro. Subsequently, Bai et al. [42] also isolated putative FGSCs from 4–6-month-old pigs, and these cells expressed similar characteristics to the mouse putative FGSCs over 4 months in in vitro culture. Interestingly, the pig putative FGSCs were mainly located in the ovarian theca rather than the ovarian cortex, where putative FGSCs have typically been found in other mammalian species. In addition, Parte et al. [43] identified very small, embryonic-like putative stem cells with a high nucleocytoplasmic ratio and pluripotent property in the OSE of adult female mammals such as rabbit, sheep, marmosets, and postmenopausal women. These cells have the potential to develop into oocyte-like structures in vitro; however, it is required to further confirm whether postnatal oogenesis is originated from putative FGSCs.

Mvh, the homolog gene of Vasa, which is acknowledged as the specific marker of reproductive cell lineage, is widely considered to be expressed in the cytoplasm rather than the cell membrane of germline cells [44–46]. For this reason, the feasibility of using Mvh as putative FGSCs sorting marker and the validity of their results have been greatly debated. In order to dispel the doubts, White et al. [47] had identified an epitope of Mvh which located in the extracellular domain of germ cellular membrane. The C-terminal domain of Mvh has been considered to be a sorting marker of germ cells via antibody recognition. In view of the fact that immunomagnetic sorting is a relatively crude cell isolation approach that cannot discriminate between viable, injured, and dead cells, White et al. modified the sorting method of flow cytometry to separate normal activated mice putative FGSCs, and using this improved method, they isolated putative FGSCs from the cortex of the human female ovary and differentiated them into oocyte-like structures in vitro for the first time. Further, putative FGSCs labeled with GFP were transplanted into ovarian cortex and xenotransplanted into immunodeficient mouse ovaries. As a result, follicles containing GFP positive oocytes eventually formed. Therefore, it can be inferred that a small number of putative GSCs exist in adult female ovaries.

Virant-Klun et al. [48] obtained a part of ovarian tissue by a laparoscopic ovarian cortex biopsy from 3 patients with premature ovarian failure, and small round cells expressing Sox-2 marker of pluripotency were observed among epithelium cells just after the OSE scraping. Primitive oocyte-like cells and typical round-shaped cell clusters were developed when the scraped cells were cultured in a medium containing follicular fluid to provide some ovarian niche. Using similar technologies, they also successfully separated a class of SSEA-4 positive cells from the OSE of normal, menopausal women and premature ovarian failure patients. Immunocytochemical and genetic analyses showed that these cells were primordial germ cells with pluripotent stem cell markers. These cells can be developed into an oocyte-like structure and expressed several oocyte-specific transcription factors. In summary, this class of SSEA-4 positive cells can be identified as putative FGSCs based on several stem cells criteria.

### 4.3. Application of Mammalian Putative FGSCs for the Construction of Transgenic Animals

To determine whether the short-term cultured mouse putative FGSCs can produce transgenic offspring, Zhang et al. [49] primarily cultured the GFP positive FGSCs for 3–5 days; then these cells were transplanted into the infertile mice ovaries. Two months later, a large number of normal follicles including GFP positive oocytes were found in the transplanted ovaries, and the infertile female mice produced the GFP positive offspring after mating with wild-type male mice. Dnaic2 is considered a primary ciliary dyskinesia-associated gene and is mainly expressed in mouse ovaries, testicles, and so forth [50], and Oocyte-G1 is a newly discovered member of the kinesin superfamily that plays an important role in organelle and protein transport [51]. Here, Zhang et al. [49] chose the Dnaic2 and Oocyte-G1 to explore the construction of transgenic mice model using putative FGSCs. Two genes were recombined with lentiviral vectors and infected into putative FGSCs, respectively. Then, the modified putative FGSCs were transplanted into the ovaries of infertile mice and mated with wild-type male mice. The results showed that the offspring of Dnaic2 transgenic female mice were genetically characterized with subfertility or infertility; meanwhile, the Oocyte-G1 transgenic offspring showed postnatal growth retardation. The experiment successfully proved the availability of the method of transgenic animal construction via putative FGSCs. Thus, Zhang et al. pioneered a new method for the construction of transgenic animals that is mediated by putative FGSCs. This method has the advantages of being faster, cheaper, and more efficient than DNA microinjection, nuclear transfer, and mutagenesis of SSCs for the construction of transgenic animals. And in the future, it may serve as a powerful tool for gene therapy, biotechnology research, and, especially, the construction of transgenic animals. In addition, Zhou et al. [41] also used rat putative FGSCs to construct a fat-1 transgenic rat that can express the traits associated with fat-1 functions. Compared with mice, rats are more closely related to human being; therefore, the rat FGSC-mediated transgenic animal model may be more suitable for the study of some clinical diseases, such as premature ovarian failure and ovary infertility.

## 5. The Prospect of Putative GSCs in Mammalian Ovaries Research

### 5.1. Challenges of Future Putative FGSCs Research

**Figure 2:**
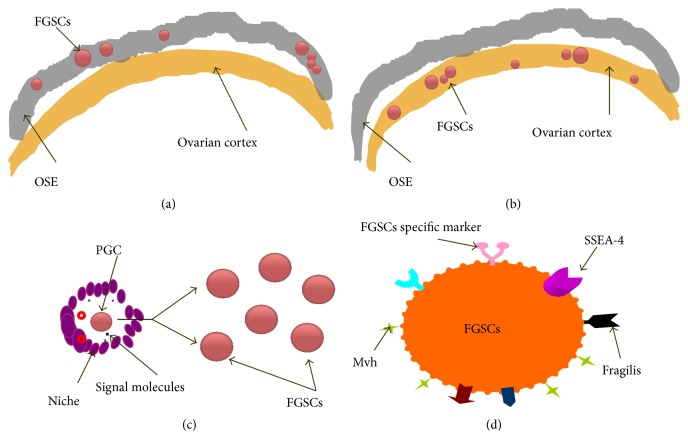
The existing challenge of putative FGSCs. ((a) and (b)) The putative FGSCs exact positioning in OSE or ovarian cortex. (c) The “niche” signals regulate PGC to differentiate into putative FGSCs. (d) Is there a putative FGSCs specific marker in cell membrane?

In recent years, with the deepening of the research, a certain amount of putative FGSCs in the postnatal mammalian ovary has been gradually accepted by the reproductive area. However, as a new research field, there are still many problems to be solved before we can truly know and apply the putative FGSCs in future. First, where is the exact location of putative FGSCs in ovary? At present, these cells have been considered to be mainly localized in the cortex or surface epithelium of the ovary, but their exact position in the ovary is still unknown (Figures 2(a) and 2(b)). Second, what is the real source of putative FGSCs? There remain a few dormant PGCs in ovary, and the microenvironment of PGCs can secrete signals to cause some PGCs transition from the dormant stage to the proliferative phase. The active PGCs can obtain the ability of self-renewal and differentiation potency. FGSCs may have originated from the active PGCs (Figure 2(c)). Third, how can a specific marker of putative FGSCs be defined? FGSCs are mainly separated and purified using germ cell-specific markers (such as Mvh) or pluripotent stem cell markers (such as SSEA-4); it is inevitably mixed with other impurity cells (such as oocyte or other cell lineage) when putative FGSCs are isolated by using these cell markers. If more specific or unique putative FSGCs marker can be discovered, it would greatly facilitate putative FGSCs identification and purification (Figure 2(d)). Fourth, what is the regulatory mechanism of the “niche” environment of putative FGSCs? The microenvironment of stem cells consists of the near cell, extracellular matrix, and some soluble factors; it is generally called “niche.” There are a few putative FGSCs existing in the mammalian ovaries, and what signals or molecules which had regulated the proliferation and differentiation of putative FGSCs are still unknown (Figure 2(c)). In addition, like other cell lines, putative FGSCs will undergo an aging process and lose their ability of propagation and differentiation. It may be of benefit to propagate and culture putative FGSCs if we can find the proliferative signaling or molecular mechanism. Finally, the transformation from experimental research to clinical application of putative FGSCs remains to be explored. The studies about putative FGSCs are mainly performed in vitro, and some problems such as putative FGSCs differentiation, primordial follicle formation, follicle development, and in particular normal ovum formation in vivo need to be solved. And even if mature eggs are produced in vivo, there are some problems against such as epigenetic, especially imprinting abnormalities which precautions need to be taken precaution during fertilization and culture to cleavage stage. In a word, there is still a long way to go for human putative FGSCs to be applied in a future medical domain.

### 5.2. The Potential Applications of Putative FGSCs Research

With the isolation and culture technology getting mature, now we can construct putative FGSCs lines from many kinds of mammals, and the establishment of these cell lines laid an important foundation for the potential application.

A main cause of some clinical diseases, such as ovarian dysfunction, infertility, and physiological or pathological premature ovarian failure, is the lack of oocytes. Like in vitro fertilization and embryo transfer (IVF-ET) technology, the patient's putative FGSCs can be obtained via primary cultivation and breeding in vitro; then, these cells are transplanted back to the patient's ovaries by microinjection to form oocytes and remedy the functional defects of ovaries. For women who had tumor, it is a pathway that putative FGSCs transplantation can be considered as preserving or recovering the fertility after radiation therapy. At present, the above technique had been successfully performed to restore the reproductive functions of infertile mice model [23, 39]. Although there is still a long way to arrive from experimental research to clinical therapy, the application of FGSCs will have a profound impact on clinical therapy strategies in various reproductive diseases.

ESCs have a great application prospect in the field of cell therapy. However, it is controversial due to the restriction of ethics and safety. There is certain tumorigenicity and low efficiency for induced pluripotent stem cells (iPSCs) to clinical application in future; thus, it is necessary to look for new source of multipotent stem cells (mGCs). Previous researches showed that SSCs can be transformed into mGCs under certain conditions in vitro and that mGCs have characteristics and differentiation similar to ESCs [52–54]. As another kind of GSCs, putative FGSCs may be taken as a new source of mGCs. Actually, Wang et al. [40] had successfully converted putative FGSCs from neonatal and prepubertal mice into female embryonic stem-like cells (fESLCs) that exhibit properties similar to ESCs. Thus, putative FGSCs have the potential to be applied to replace ESCs and iPSCs in cellular therapy. There are many significant advantages for putative FGSCs such as avoiding using embryos as a source of cells, not involving viral vectors, and not requiring reprogram. In addition, the genetic modification animal model can be built through inducing target gene into putative FGSCs. Finally, putative FGSCs are of great significance to the preservation of endangered species and provide source of cells for reproductive related drug screening.

In a word, FGSCs possess broad application prospects such as regenerative medicine, biological engineering, and animal breeding. Although the discovery of putative FGSCs starts relatively late and is deficient in practice, as a kind of new stem cells, it will provide us with the broad application prospects, and humans will obtain huge benefits from the application of putative FGSCs in the future.
